# A Novel Multimodal Digital Service (Moderated Online Social Therapy+) for Help-Seeking Young People Experiencing Mental Ill-Health: Pilot Evaluation Within a National Youth E-Mental Health Service

**DOI:** 10.2196/17155

**Published:** 2020-08-13

**Authors:** Mario Alvarez-Jimenez, Simon Rice, Simon D'Alfonso, Steven Leicester, Sarah Bendall, Ingrid Pryor, Penni Russon, Carla McEnery, Olga Santesteban-Echarri, Gustavo Da Costa, Tamsyn Gilbertson, Lee Valentine, Laia Solves, Aswin Ratheesh, Patrick D McGorry, John Gleeson

**Affiliations:** 1 Orygen Parkville Australia; 2 Centre for Youth Mental Health The University of Melbourne Melbourne Australia; 3 The School of Computing and Information Systems The University of Melbourne Melbourne Australia; 4 headspace National Youth Mental Health Foundation Melbourne Australia; 5 School of Culture and Communications The University of Melbourne Melbourne Australia; 6 Centre for Mental Health Swinburne University of Technology Hawthorn Australia; 7 Hotchkiss Brain Institute Psychiatry Department University of Calgary Calgary, AB Canada; 8 Consorci Sanitari del Maresme Department of Psychiatry Hospital of Mataro Mataro Spain; 9 School of Behavioural and Health Sciences Australian Catholic University Melbourne Australia; 10 Healthy Brain and Mind Research Centre Australian Catholic University Melbourne Australia

**Keywords:** mHealth, youth, social media, social networking, mobile phone, internet-based intervention

## Abstract

**Background:**

Mental ill-health is the leading cause of disability worldwide. Moreover, 75% of mental health conditions emerge between the ages of 12 and 25 years. Unfortunately, due to lack of resources and limited engagement with services, a majority of young people affected by mental ill-health do not access evidence-based support. To address this gap, our team has developed a multimodal, scalable digital mental health service (Enhanced Moderated Online Social Therapy [MOST+]) merging real-time, clinician-delivered web chat counseling; interactive user-directed online therapy; expert and peer moderation; and peer-to-peer social networking.

**Objective:**

The primary aim of this study is to ascertain the feasibility, acceptability, and safety of MOST+. The secondary aims are to assess pre-post changes in clinical, psychosocial, and well-being outcomes and to explore the correlations between system use, perceived helpfulness, and secondary outcome variables.

**Methods:**

Overall, 157 young people seeking help from a national youth e-mental health service were recruited over 5 weeks. MOST+ was active for 9 weeks. All participants had access to interactive online therapy and integrated web chat counseling. Additional access to peer-to-peer social networking was granted to 73 participants (46.5%) for whom it was deemed safe. The intervention was evaluated via an uncontrolled single-group study.

**Results:**

Overall, 93 participants completed the follow-up assessment. Most participants had moderate (52/157, 33%) to severe (96/157, 61%) mental health conditions. All a priori feasibility, acceptability, and safety criteria were met. Participants provided mean scores of ≥3.5 (out of 5) on ease of use (mean 3.7, SD 1.1), relevancy (mean 3.9, SD 1.0), helpfulness (mean 3.5, SD 0.9), and overall experience (mean 3.9, SD 0.8). Moreover, 98% (91/93) of participants reported a positive experience using MOST+, 82% (70/93) reported that using MOST+ helped them feel better, 86% (76/93) felt more socially connected using it, and 92% (86/93) said they would recommend it to others. No serious adverse events or inappropriate use were detected, and 97% (90/93) of participants reported feeling safe. There were statistically significant improvements in 8 of the 11 secondary outcomes assessed: psychological distress (*d*=−0.39; *P*<.001), perceived stress (*d*=−0.44; *P*<.001), psychological well-being (*d*=0.51; *P*<.001), depression (*d*=−0.29; *P*<.001), loneliness (*d*=−0.23; *P*=.04), social support (*d*=0.30; *P*<.001), autonomy (*d*=0.36; *P*=.001), and self-competence (*d*=0.30; *P*<.001). There were significant correlations between system use, perceived helpfulness, and a number of secondary outcome variables.

**Conclusions:**

MOST+ is a feasible, acceptable, and safe online clinical service for young people with mental ill-health. The high level of perceived helpfulness, the significant improvements in secondary outcomes, and the correlations between indicators of system use and secondary outcome variables provide initial support for the therapeutic potential of MOST+. MOST+ is a promising and scalable platform to deliver standalone e-mental health services as well as enhance the growing international network of face-to-face youth mental health services.

## Introduction

### Background

Mental ill-health is the number one cause of disability worldwide [1] and accounts for 8 million deaths annually [2]. Mental illness strikes the young, with 75% of all mental disorders having their onset before the age of 25 years [3], and for many, it follows a relapsing course [4,5]. The timing and course of mental illness disrupts the attainment of critical developmental milestones for young people, such as completing their education, entering the job market, and developing intimate relationships [6]. This can result in devastating lifelong consequences, including increased risk of chronic unemployment, lower income and living standards, homelessness, social isolation, suicide, and early mortality [7-9].

Despite the prevalence and impact of mental illness, between 35% and 57% of people with mental health disorders do not access treatment in high-income countries [1]. The corresponding range for low- and middle-income countries is 76% to 85% [1]. The mismatch between the prevalence of mental illness and the rate at which treatment is accessed is the greatest for young people. The most recent Australian national survey of young people’s mental health revealed that only 13% of men and 31.2% of women aged 16-24 years who had experienced a mental disorder in the preceding 12 months received professional help [10]. The reasons for low rates of treatment access among young people include low help-seeking due to fear of stigma and embarrassment, confidentiality concerns, negative prior experiences of treatment, poor mental health literacy, lack of knowledge of available resources, a preference for self-reliance, and social isolation [11-13]. Additional structural and logistical barriers include geographical distance, poverty, out-of-pocket expenses associated with treatment, and lack of availability of services [14].

The internet, mobile technologies, and social media have the potential to address the global crisis in the rate at which young people access evidence-based mental health care. Internet-enabled mobile devices are a pervasive element of young people’s lives, with 45% of adolescents being on the web almost constantly [15]. Social media has become a key vehicle for young people to communicate with one another. Almost all young people have at least one active social media account, with over 70% using social media multiple times a day—a rate that has doubled between 2012 and 2018 [16]. Recent surveys demonstrate that young people experiencing mental ill-health are also avid users of social media: 97% use it regularly, 2.6 hours per day on average [17]. Particularly relevant to the clinical potential of social media, engaging with peers online about mental health issues increases the likelihood of seeking professional support [18], and many young people use social media to obtain mental health information [19]. Similarly, initial studies showed that 74% of young people who experience mental ill-health would like to obtain help from mental health clinicians via social media [17] and value web-based services run by professionals [20]. Thus, social media offers a unique opportunity to provide and boost web-based youth mental health interventions.

Currently, there are 4 main types of digital interventions for mental health: self-guided web-based interventions, standalone mental health mobile apps, online peer support groups or interventions, and web-based counseling with registered professionals. Previous trials have shown that the first generation of self-guided web-based interventions, particularly those delivering cognitive behavioral therapy (CBT) and including human support [21], are effective in alleviating anxiety and depressive symptoms [22,23]. However, the impact of these web-based interventions has been hindered by two constraints. First, these interventions were designed to *mimic* face-to-face interventions, resulting in high attrition rates (particularly for interventions with no human support) and little treatment innovation (eg, novel intervention models harnessing mobile technology to provide intensive, real-time support) [24]. Second, these interventions were developed as *separate products* [24], with little consideration as to how they fit in with existing clinical services, resulting in an overall lack of integration of web-based support with clinical services [25].

The number of mobile apps targeting mental health has grown exponentially over the past few years. According to a 2017 report, almost 500 unique apps were targeting mental health disorders in 2017 [26]. More recently, a systematic search of mobile apps focused on wellness and stress management found over 1000 publicly available apps [27]. However, although mental health apps are flooding the consumer market, very few studies have examined their effectiveness [28], and many apps do not follow evidence-based guidelines and principles [29]. For example, of the apps focused on psychosocial wellness and stress management, only 1% involved therapist support, and less than 2% were designed to supplement in-person treatment and 2% had any research publications (with most of these being a single feasibility or efficacy study) [27]. Examining the existing evidence, two meta-analyses found significant but small effects for reductions in anxiety [30] and depression [31] from smartphone interventions as compared with control conditions. However, a more recent meta-analysis including only mobile apps designed to treat mental health symptoms found small significant effects on depression, with no significant effects on anxiety, suicidal ideation, self-injury, or alcohol use [32]. It must be noted that the effects of mental health apps (g=−0.14 to 0.18) were significantly smaller than those of web-based mental health interventions [23,33-35] (g=0.84-1.09). These smaller effects may be explained by the lack of human support in most mental health apps; poor adherence, which is considered to be a major pitfall of mental health apps [36], or low use of key app features; and possibly a lack of sustained and structured attention to content and features that may be needed for an intervention to yield significant benefits [32]. To date, most mobile mental health apps have been designed in academic labs or by commercial companies, usually outside of clinical services, resulting in an overall lack of integration with existing clinical services [37].

A growing number of online peer support groups and social networking sites (SNS) exist for people with mental health problems. Overall, the extant evidence suggests that online peer support groups can foster a sense of social connectedness, empowerment, and improved quality of life as well as reduce depression and emotional distress [38-40]. That said, the type and function of online peer support groups and SNS appear to have a significant effect on their outcomes. For example, unmoderated forums and SNS can lead to increased contagion, distress, and collusion among users [39]. Conversely, SNS interventions that have been moderated, ideally by professionals, have been found to be safe, engaging, supportive, and useful [39,41-44]. This is in keeping with the findings that young people with mental ill-health have a strong preference for purpose-built, moderated, social media–based interventions enabling peer-to-peer contact as well as clinician support [45]. Despite this emerging evidence, the majority of available peer support groups do not provide professional moderation and clinical support or incorporate evidence-based, user-directed web-based therapy.

The third main type of digital support is web-based counseling (ie, real-time web chat with clinicians). There is initial evidence that web chat is an effective way to deliver mental health support [46], with recent trials showing comparable levels of effectiveness [47], therapeutic alliance [48], and perceived helpfulness with face-to-face therapy [48]. Recent studies also indicate that young people are increasingly turning to web chat to receive mental health support [49]. However, these web-based services are also constrained by capacity and scalability due to their reliance on one-to-one web-based support.

Our group has developed a novel and evolving model of web-based behavioral interventions entitled Moderated Online Social Therapy (MOST). The MOST model merges (1) interactive web therapy, (2) peer-to-peer web-based social networking, (3) peer, and (4) clinical moderation. Successive iterations and evolutions of MOST have been successfully adapted for, and trialed with, young people with psychosis [42,50], at clinical risk of psychosis [43], suicidal risk [51], depression [44,52], and social anxiety [53], as well as relatives of young people with psychosis [54] and depression [55]. To respond to the crisis in access to care by young people who experience mental ill-health, our group has developed a new model of web-based clinical support entitled Enhanced Moderated Online Social Therapy (MOST+). MOST+ fully merges the effective components of web-based interventions, online peer support groups, and web-based counseling into a single platform. As such, MOST+ combines (1) a wide range of evidence-based, interactive, user-directed web-based interventions; (2) secure and supportive peer-to-peer web-based social networking; (3) peer moderator support; (4) clinical moderation; and (5) on demand web chat with registered clinicians. Thus, MOST+ was designed to deliver an accessible and scalable web-based mental health service catering to the varying needs and preferences of young people by flexibly integrating multiple modes of effective web-based support.

### Objectives

The overarching aim of this study (trial registration: ACTRN12617000370303) was to determine the feasibility, acceptability, and safety of MOST+ for young people seeking online mental health support. The secondary aims of the project were (1) to assess changes in psychological distress, well-being, depression, stress, social support, loneliness, basic psychological needs (self-competence, relatedness, and autonomy), strengths usage, and mindfulness skills from the point of engagement to post intervention and (2) to explore the associations between system usage, perceived helpfulness, and secondary outcome variables. We hypothesized that MOST+ would be regularly used, favorably received, and safe against a priori established criteria (described in detail in the *Results* section) [56].

## Methods

### Study Design and Setting

The methods of this study have been described in detail elsewhere [56]. Briefly, this study employed an uncontrolled single-group design [57]. The MOST+ intervention was embedded within eheadspace services, a national web counseling service funded by the federal government for young people aged 12 to 25 years in Australia [49]. eheadspace is staffed by qualified and supervised mental health clinicians providing synchronous web chat, email support, and telephone-based mental health support. For this study, 27 eheadspace clinicians were trained to deliver online support using the MOST+ platform. Two of these clinicians, based at the eheadspace operation center, provided web chat support via MOST+ from 4 PM to 12 AM every day. They were registered with mental health clinicians with prior specialist training and experience in the delivery of e-mental health support to young people in distress. The MOST+ platform was available for a period of 9 weeks, including a 5-week recruitment period (ACTRN12617000370303).

### Participants and Recruitment

The sample included 157 young people recruited via an opt-in process at the point of entry to eheadspace through a link on the home page. The inclusion criteria were as follows: (1) help-seeking young people with concerns about their own mental health, (2) people aged 16 to 25 years, and (3) people with the ability to provide informed consent and comply with study procedures. The participants who indicated on the web that they understood and consented to the study procedures were recruited into the study.

To ensure the safety of the online social network, some participants were excluded from access to the social networking component of MOST+ (defined as partial access; see under *Web Counseling*). These exclusions were (1) acute risk of self-harm requiring urgent intervention (ie, suicidal ideation with a current plan and intent) indicated by a young person on web chat or by endorsing predetermined screening questions; (2) participation in the MOST+ social network was deemed likely to interfere with appropriate clinical management of mental health symptoms (eg, psychosis) or increase the risk of harm to self or others by an eheadspace clinician; and (3) inability to confirm age and conduct induction via a research assistant telephone contact (Multimedia Appendix 1). Participants with partial access could access all other components of MOST+ (ie, web chat and interactive web-based therapy). This design feature enabled us to compare outcomes across these 2 levels of access.

The mean age of the participants was 19.1 (SD 2.3) years, with 77% female participants. A total of 87% (137/157) of the participants were born in Australia and 11% (17/157) spoke languages other than English. Moreover, 70% (110/157) of the participants were from metropolitan areas, 28% (44/157) from rural areas, and 2% (3/157) from remote areas. In addition, 3% (5/157) of the participants identified themselves as Aboriginals and/or Torres Strait Islanders. Furthermore, 59% (93/157) of the participants had not previously used youth mental health services and 37% (58/157) had never received any mental health support. A total of 57% (89/157) of the participants were engaged in paid work and 77% (121/157) were studying part-time or full-time. The main reasons for seeking help included sadness (38%: 60/157) and anxiety (22%:35/157), followed by feelings of distress (9.6%:15/157). Baseline clinical measures indicated that the majority of participants had mental ill-health. Specifically, the mean baseline Kessler 10 (K10) score was 32.03 (SD 7.72), with 61% (57/93) scoring 30 (indicative of a severe mental health disorder) and 33% (31/93) scoring 25-29 (indicative of a moderate mental health disorder) [58] (Table 1). Similarly, the mean baseline Patient Health Questionnaire-9 (PHQ-9) score (measuring depression) was 15.76 (SD 6.32), with 59% (55/93) scoring 15 (indicative of moderately severe depression) and 24% (22/93) scoring 10-14 (indicative of moderate depression) [59] (Table 1 [60-71]).

**Table 1:** Mean (SD) and within-group effect sizes (Cohen d) for outcome measures (N=93).

Characteristics	Baseline, mean (SD)	Follow-up, mean (SD)	*P* values	Cohen *d* (95% CI)
K10^a^	32.03 (7.680)	29.43 (8.119)	<.001	−0.39 (−0.68 to −0.10)
WEMWS^b^	6.58 (2.174)	7.60 (2.232)	<.001	0.51 (0.21 to 0.80)
PSS^c^	10.65 (2.483)	9.52 (2.940)	<.001	−0.44 (−0.72 to −0.14)
PHQ-9^d^	15.76 (6.322)	13.98 (6.514)	.008	−0.29 (−0.57 to −0.01)
UCLA^e^	9.23 (1.984)	8.83 (2.224)	.04	−0.23 (−0.52 to −0.06)
Competence^f^	20.69 (6.449)	22.27 (6.494)	.005	0.30 (0.01 to 0.60)
Relatedness^g^	35.61 (8.900)	36.85 (7.412)	.08	0.17 (−0.12 to 0.46)
Autonomy^h^	25.68 (6.663)	27.61 (7.148)	.001	0.36 (0.07 to 0.65)
FS^i^	10.06 (5.303)	11.28 (4.935)	.004	0.30 (0.01 to 0.59)
SUS^j^	54.20 (16.621)	56.40 (17.361)	.21	0.13 (−0.15 to 0.42)
FMI^k^	28.77 (6.513)	30.08 (7.184)	.08	0.20 (−0.10 to 0.48)

^a^K10: Kessler 10.

^b^WEMWS: 3 items from the Warwick-Edinburgh Mental Well-being Scale.

^c^PSS: Perceived Stress Scale.

^d^PHQ-9: Patient Health Questionnaire-9.

^e^UCLA: UCLA Loneliness Scale (Version 3).

^f^Competence: subscale of the Basic Psychological Need Satisfaction Scale.

^g^Relatedness: subscale of the Basic Psychological Need Satisfaction Scale.

^h^Autonomy: subscale of the Basic Psychological Need Satisfaction Scale.

^i^FS: Friendship Scale.

^j^SUS: Strengths Use Scale.

^k^FMI: Freiburg Mindfulness Inventory-Short Form.

### Intervention: MOST+

A large multidisciplinary team of researchers, clinical psychologists, programmers, creative writers, graphic artists, and experts in human-computer interaction worked in collaboration with end users to iteratively develop the MOST+ platform [42,45]. MOST+ merged (1) interactive user-directed web-based therapy (*Steps*), (2) peer-to-peer online social networking, (3) peer moderator support, (4) expert moderation, and (5) on demand web chat with clinicians.

MOST+ was conceived as an accessible web-based youth mental health service delivering immediate, short-term, flexible, and evidence-based support to help-seeking young people with mental ill-health. MOST+ was designed to be scalable through the integration of multiple modes of web-based support, thus enabling varying levels of direct support by peer moderators and clinicians.

### MOST+ Intervention Components

#### Interactive, User-Directed Psychosocial Interventions

MOST+ adopted a strengths-based approach [42] through which users were guided and prompted to identify (via an interactive card-sort game) and exercise their core personal strengths to foster psychological well-being, including social connectedness and self-efficacy. Each strength included behavioral prompts or *do its* designed to support young people in applying their core strengths (eg, courage) for specific purposes (eg, dealing with social anxiety).

**Figure 1:**
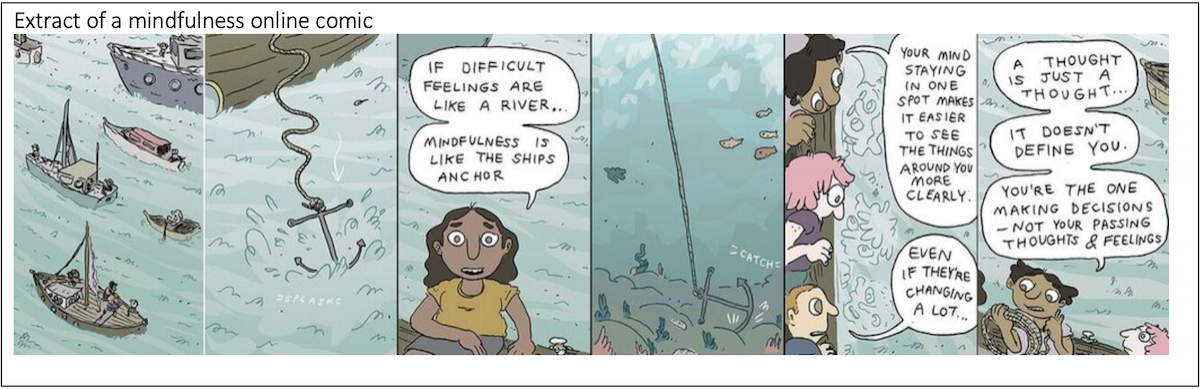
Extract of a mindfulness online comic.

Psychosocial interventions in MOST+ took the form of brief web-based comics called *steps*. Comics have been used in physical health interventions as a nonthreatening, easy-to-understand medium for patient education [72]. We chose to use comics in MOST+ because of their ability to merge persuasive metaphors and character-driven narratives [73], potentially making therapeutic concepts more accessible, engaging, and compelling for young people. Comics were developed by clinical psychologists, professional novelists, and comic artists in partnership with young people (Figure 1). This process included focus groups with young people to identify salient therapeutic themes as well as continual feedback and co-design sessions involving artists, novelists, psychologists, and young people across all phases of comic development (from initial scripting to comic drawing and coloring) [74,75]. MOST+ included 52 discrete, interactive therapy comics designed to address the main concerns for helping young people to access web counseling at eheadspace, including managing immediate distress, low mood, anxiety, social anxiety, relationship issues, and difficulties at school or work [49]. The comics were informed by evidence-based psychological therapies including CBT [76-78], mindfulness [79,80], self-compassion [81], and positive psychology interventions [82]. The comics were designed to be race, age, and gender neutral, with the purpose of maximizing their cultural, sexuality, and gender acceptance. The steps further incorporated *do its* to help participants exercise therapeutic skills (eg, empathy) in a real-life context (eg, school). Young people were able to store any *do it* they wanted to complete in the future in a *playlist*. Finally, the participants had the ability to rate, like, comment on, and share any step or *do it* with others via the social networking newsfeed.

#### Web-Based Social Networking

Participants with full access were able to communicate with one another and the peer moderators in the *Café* and *Talk it Out* sections to foster social support and problem-solving skills. The Café included a newsfeed where participants and moderators were able to create posts to share thoughts, information, pictures, and videos and respond to other users’ posts by commenting, *liking,* or *reacting* to content. The *reactions* were designed to facilitate social support (eg, *I get you* and *thinking of you*). Each participant could personalize their own profile with images and a bio and could visit the *wall* of fellow users, where their posts and general activities were displayed.

*Talk it Out* was designed as a collaborative problem-solving feature informed by the evidence-based problem-solving framework [83,84]. Participants could suggest topics (eg, how to make new friends at a new school in year 11?) and discuss solutions with trained peer moderators and other young people. Peer moderators encouraged young people to define the problem, brainstorm possible solutions, identify the pros and cons, and summarize possible solutions. Previous problems and group solutions were stored in the system, providing an easily accessible solution wiki for all young people.

#### Web Counseling

The web chat was fully integrated within MOST+. Young people using the system could request access to a clinician-delivered web chat between 4 PM and 12 AM. This included real-time web counseling focused on reducing immediate distress, supporting positive self-care, and facilitating referral to additional support where appropriate. Following a web chat session and based on the context of the consultation, MOST+ clinicians suggested specific, relevant content from MOST+ (eg, web-based comic, *Talk it Out*, or *do it*) to the young person. These suggestions appeared in the users’ notifications sections as well as in the chat window. Web chats were classified based on the level of risk, with chat requests indicating that suicidal ideation and psychotic symptoms were prioritized.

### Partial Versus Full Access to MOST+

Participants who consented to the study completed a 15-min web-based survey [56]. Following this survey, all young people were granted partial access to MOST+ consisting of real-time, clinician-delivered web chat as well as user-directed psychosocial interventions. Full access to MOST+, which additionally included peer-to-peer web-based social networking, was granted based on a three-tiered screening process designed to ascertain the safety and appropriateness of this component for each young person (Multimedia Appendix 1).

### Duration of Access to MOST+

Irrespective of the level of access, all participants were enrolled in the MOST+ intervention for 1 week, with the option to extend their enrollment on a weekly basis over the duration of the intervention period (ie, a minimum of 1 week to a maximum of 9 weeks). The participants were shown a *count-down watch* depicting the time left before their account deactivation. Following account deactivation, all information and activity pertaining to the participant’s account (eg, profile, posts) were hidden from the MOST+ system. However, the participants were able to reactivate an expired account at any time during the study intervention period. Multimedia Appendix 2 provides examples of the participants’ enrollment timelines, with each colored line representing a different potential timeline over the duration of the pilot. This feature was implemented to protect the privacy of the large group of one-time users of eheadspace services [49] while ensuring a lively and safe online social network for regular users. Specifically, based on the patterns of usage of eheadspace, we anticipated that a significant proportion of users would only use MOST+ for a short period. By automatically hiding their activity following their short-term use of MOST+, we aimed to protect their privacy in the long term, while maintaining the currency and dynamism of the social network and facilitating the safety management of the social network at scale.

### Moderation

MOST+ incorporated clinician as well as peer moderation. Clinical moderation primarily focused on ensuring the safety of the social network. Specifically, MOST+ clinicians monitored new contributions to the network for indicators of clinical risk. The social network was moderated by an on-duty MOST+ clinician daily. Safety checks (ie, monitoring any indications of risk on the social network) were undertaken a minimum of 2 times per week day and once daily on weekends and public holidays (see *Safety Protocol* details below). The clinicians were regularly supervised throughout the trial by their usual eheadspace shift supervisors.

The *Cafe* was led and moderated by trained young people with the experience of having lived with mental illness (*Peer workers*). Peer workers were peer moderators who facilitated social learning using MOST+ in desired ways (eg, self-disclosing, using therapy content to deal with difficulties) [85]. Drawing on the growing evidence for peer support having a positive impact on the levels of hope, empowerment, and quality of life [86], peer workers also provided general guidance and peer-to-peer support. In addition, peer workers guided the problem-solving discussions in *Talk it Out* and posted links to relevant therapeutic resources and *tips*. Finally, peer workers seeded discussion threads and *icebreakers* to enable useful, enjoyable conversations and facilitate meaningful relationships. Peer moderators were supervised weekly by members of the research team (clinical psychologists and peer support coordinators).

### Safety Protocol

The safety protocol comprised 3 levels of security: (1) system and privacy protection, (2) web safety, and (3) clinical safety. MOST+ had built-in security and data protection to prevent unauthorized access to the platform, which has been described elsewhere [56]. These measures conform to industry best practices as defined by the Open Web Application Security Project. Privacy and web safety were managed in accordance with the Australian Communications and Media Authority. Specifically, the participants were informed of, and were required to accept, the terms of use of MOST+, which included clauses on protecting their privacy and that of others as well as guidelines on proscribed behavior (ie, disrespectful behavior or offensive comments).

The MOST+ clinical safety protocol included manual and automated procedures. First, information related to clinical risk (posts or messages) was screened by clinical moderators twice each weekday and daily on weekends and public holidays. Second, MOST+ incorporated an automatic alert system that monitored self-harm–related terms posted on the social feed. Any detected increased risk or inappropriate use activated the safety protocol (Multimedia Appendix 3). Finally, a *report function* enabled the participants to alert the moderators to inappropriate use of the system (eg, discriminatory comments posted on the social network).

### Participants’ Data Management

The participants were able to control the extent to which they could be identified by other users within the social network, including whether they used their first name or a nickname and whether their profile picture included a photo. As noted above, following account deactivation, the participants’ accounts and activities were hidden from MOST+. The participants could also choose to *switch off* their profiles to hide all past posts and comments and anonymize their contributions to *Talk it Out*. The participants were informed that any records of user activity hidden from the social network were retained by the researchers for the purpose of analysis. Specifically, all user-generated data were encrypted and retained in the MOST+ database throughout the trial. Upon completion of the trial, MOST+ was decommissioned and the database was exported and stored in a deidentified format on an Orygen server for research purposes.

### Outcome Measures

The primary outcome variables were intervention feasibility, acceptability, and safety. All outcomes were assessed at baseline and at follow-up. Baseline assessments were conducted on the web as part of the onboarding process. Follow-up assessment occurred approximately 4 days after the initial account deactivation (ie, 4 days after a participant opted not to renew their account for an additional week). For those participants who maintained active enrollment across the intervention period, follow-up occurred as soon as possible following the conclusion of the pilot. The participants received a short message service notification indicating that their web follow-up survey was due and that they were able to complete survey items either via the web or telephone.

A self-report user feedback questionnaire was developed based on the user experience approach [87] assessing the following themes: (1) acceptability, (2) helpfulness, and (3) safety. Acceptability was determined against the following a priori indicators: (1) participants provided ratings of the MOST+ platform averaging above three out of five across feedback questions regarding ease of use, relevancy, helpfulness, and overall experience; (2) at least 60% (56/93) of the participants reported that the MOST+ intervention provided relevant and helpful support; and (3) at least 80% (74/93) of the participants would recommend MOST+ to other young people experiencing similar difficulties (Table 2). In addition, the MOST+ intervention was considered safe if (1) at least 90% (84/93) of the participants reported the web-based intervention to be safe, (2) none of the participants experienced a serious adverse event as a result of their engagement with the system, and (3) there were no unlawful entries into the MOST+ system detected by study programmers during the 8-week pilot.

Secondary outcome measures included self-report measures of psychological distress, well-being, depression, stress, social support, loneliness, basic psychological needs (self-competence, relatedness, and autonomy), strengths usage, and mindfulness skills (Table 3).

**Table 2:** Acceptability, safety, and perceived helpfulness ratings using Enhanced Moderated Online Social Therapy (N=93).

Questions		Mean (SD)	Median	Values, n (%)^a^	
**Overall acceptability**					
	How would you describe your overall experience on MOST+^b^?^c^	3.9 (0.8)	4		91 (98)
	Please rate the helpfulness of using MOST+^d^	3.5 (0.9)	4		80 (86)
	Please rate how quickly you were able to find what you needed on MOST+ (ease of use)^e^	3.7 (1.1)	4		80 (86)
	Please rate how relevant you found the content on MOST+^f^	3.9 (1.0)	4		82 (88)
**Safety and support**					
	Has using MOST+ helped you to better access support from others?^g^	3.59 (1.125)	4		79 (85)
	Please rate whether using MOST+ helped you feel better^h^	3.38 (1.03)	3		76 (82)
	Please rate whether using MOST+ helped you feel more socially connected^h^	3.18 (1.15)	3		70 (86)
	Please rate whether you felt safe using MOST+^i^	4.43 (0.82)	5		90 (97)

^a^Number of cases responding in the positive range (3 or higher) based on complete responses.

^b^MOST+: Enhanced Moderated Online Social Therapy.

^c^Items rated from 1=not at all positive to 5=very positive.

^d^Items rated from 1=not at all helpful to 5=very helpful.

^e^Items rated from 1=not at all quickly to 5=very quickly.

^f^Items rated from 1=not at all relevant to 5=very relevant.

^g^Items rated from 1=not at all to 5=very much.

^h^Items rated from 1=not at all safe to 5=very safe.

^i^Items rated from 1=not at all confidential to 5=very confidential, asked of participants will full access only.

**Table 3:** Overview of secondary outcomes and measures used.

Outcomes of interest	Measures	Descriptions
Psychological distress	K10^a^	10-item, widely recommended measure of psychological distress; validated in adolescents [1]
Psychological well-being	WEMWS^b^	3 items of the WEMWS are included in the eheadspace Minimum Data Set and assessed in this study: “I’ve been interested in new things,” “I’ve been feeling useful,” and “I’ve been feeling good about myself” [2]
Perceived stress	PSS^c^	10-item measure of the degree to which situations in one’s life are appraised as stressful. Widely used, with acceptable psychometric properties [3]
Depression	PHQ-9^d^	9-item measure of severity of depression. Validated in psychiatric and primary care populations [4,5]
Loneliness	UCLA^e^	20-item measure assessing how often the respondent feels disconnected from others. Highly acceptable reliability and validity [6]
Basic psychological needs of competence, relatedness, and autonomy	BPNS^f^	21-item measure with 3 subscales (competence, autonomy, and relatedness), drawing from self-determination theory [7]. Widely used [8,9]
Social support	FS^g^	6-item measure of perceived social isolation, with acceptable psychometric properties in the older adult population [10]
Strengths use	SUS^h^	14-item measure assessing the extent to which respondents use their strengths, drawing from positive psychology literature [11]
Mindfulness skills	FMI^i^	14-item measure of mindfulness. Appropriate for use in contexts where little experience or knowledge of mindfulness can be expected. Acceptable reliability and validity, including in clinical samples [12]

^a^K10: Kessler 10.

^b^WEMWS: 3 items from the Warwick-Edinburgh Mental Well-being Scale.

^c^PSS: Perceived Stress Scale.

^d^PHQ-9: Patient Health Questionnaire-9.

^e^UCLA: UCLA Loneliness Scale (Version 3).

^f^BPNS: Basic Psychological Need Satisfaction Scale.

^g^FS: Friendship Scale.

^h^SUS: Strengths Use Scale.

^i^FMI: Freiburg Mindfulness Inventory-Short Form.

### Analyses

The patterns of intervention use were tracked in real time. Aggregated data from the user feedback questionnaire were compared with the a priori acceptability and safety criteria to determine the success of the pilot. Paired samples *t* tests were conducted and within-group effect sizes (Cohen *d*) were reported for changes in the baseline and posttest study measures. To estimate Cohen *d*, Morris and DeShon’s [88] equation was applied to correct for dependence among the means in within-group designs. Parametric and nonparametric correlations were performed as appropriate to explore the associations between the usage of MOST+, acceptability ratings, and changes in secondary outcome measures.

## Results

### Feasibility, Acceptability, and Safety

A total of 93 of the 157 participants recruited for the study were contactable and assessed at follow-up. There were no statistically significant differences in any baseline demographic or clinical variables between those who completed the follow-up assessment and those who were lost to follow-up. All a priori indicators of acceptability were met (Table 2). The participants provided positive ratings of their experience using MOST+, with mean scores of 3.5 or more (out of 5) on each of the core domains of ease of use (mean 3.7, SD 1.1), relevancy (mean 3.9, SD 1.0), helpfulness (mean 3.5, SD 0.9), and overall experience (mean 3.9, SD 0.8). In addition, 98% (91/93) of participants reported a positive experience using MOST+, 86% (80/93) considered it easy to use, 88% (88/93) reported that MOST+ was relevant to their needs, 86% (80/93) considered it helpful, 82% (76/93) reported that using MOST+ helped them feel better, 86% (70/93) felt more socially connected using it, and 92% (86/93) said that they would recommend it to other young people experiencing similar difficulties. Moreover, 46% (72/157) of the participants had full access to MOST+ and 53% (83/157) had partial access (ie, excluded from the web-based social networking). The reasons for partial access included high clinical risk either detected by the system (automatically triggering a web chat; 18%: 28/157 (of all participants) or based on the clinician’s assessment (4%: 6/157) and not being able to contact eligible participants to verify age (31%: 49/157). Participants with full access to MOST+ reported a significantly more positive overall experience (mean 4.1, SD 0.7) compared with those with partial access (mean 3.7, SD 0.8; two-tailed *t(*91)=−2.89; *P*<.001). The follow-up retention rate was also significantly higher in participants with full access (57/73, 78%) than in those with partial access (43%; *X^2^_155_*=20.1; *P*<.001). There were no other significant differences in any demographic or outcome variable at baseline or acceptability ratings at follow-up between participants with full versus partial access.

A priori set safety criteria were also met. Specifically, no adverse events, inappropriate use, reports by participants, or unlawful entries pertaining to MOST+ were detected during the study. A total of 97% (90/93) of the participants reported feeling safe using MOST+. Moreover, all clinical measures showed a trend toward improved clinical status at follow-up (Table 1). There were no significant differences in safety indicators between those with full versus partial access.

Regarding the overall use of MOST+, there were a total of 1058 log-ins during the 9-week study, with 45.2% (71/157) logging in once, 14% (22/157) logging in twice, and 40.8% (64/157) logging in 3 or more times (Table 4). A total of 585 *steps* and 244 *do its* were completed during the study. All indicators of system usage were significantly higher in those with full access to MOST+ compared with those with partial access (Table 5). In terms of the duration of access to MOST+, 66.2% (104/157) of participants did not extend their initial default period of 7 days of enrollment, 14% (22/157) requested one extension, and 19.7% (31/157) requested 2 or more extensions. Finally, 41% (64/157) of participants did not request any chats with eheadspace clinicians, 34% (53/157) requested 1 chat, and 24% (38/157) requested 3 or more chats (including automatic web chats triggered by screening items indicating possible acute risk on initial registration into MOST+; Multimedia Appendix 1).

**Table 4:** Log-ins and individual usage of the main components of Enhanced Moderated Online Social Therapy (N=157) during the pilot study.

Full sample	Characteristics		
Site component	Mean (SD)	Range	Percentage, n (%)
Log-ins	6.74 (15.21)	1-103	86 (54.8^a^)
Posts and comments	1.14 (4.69)	0-45	21 (14^b^)
Steps	3.73 (9.88)	0-87	78 (49.7^c^)
“Do its”	1.55 (6.53)	0-74	49.9 (31.8^d^)

^a^Percentage of participants with more than 2 log-ins.

^b^Percentage of participants with more than 1 posts/comments.

^c^Percentage of participants completing more than 1 step.

^d^Percentage of participants completing more than 1 *do it*.

**Table 5:** Comparison of log-ins and individual use of the main components between participant groups with full access (n=73) and participant group with partial access (n=84).

Variables	Participants with full access (n=73)			Participants with partial access (n=84)				
	Mean (SD)	Range	Participants, n (%)	Mean (SD)	Range	Participants, n (%)	*t* test (df)	*P* value
Log-ins	12.34 (18.75)	1-103	58 (79.5^a^)	1.87 (2.23)	1-18	30 (33.3^a^)	−5.10 (155)	<.001
Post and comments	2.46 (6.66)	0-45	22 (30.1^b^)	N/A^e^	N/A	N/A	N/A	N/A
Steps	6.52 (13.55)	0-87	49 (67.1^c^)	1.30 (3.35)	1-23	29 (34.5^c^)	−3.41 (155)	.001
“Do its”	2.78 (9.36)	0-74	31 (42.5^d^)	0.49 (1.30)	0-7	19 (22.6^d^)	−2.22 (155)	.03

^a^Percentage of participants with more than 2 log-ins.

^b^Percentage of participants with more than 1 posts/comments.

^c^Percentage of participants completing more than 1 step.

^d^Percentage of participants completing more than 1 *do it*.

^e^N/A: not applicable.

### Secondary Outcome Variables

There were statistically significant improvements between baseline and follow-up assessments, with a small to medium size, in psychological distress (*d*=−0.39; *P*<.001), perceived stress (*d*=−0.44; *P*<.001), psychological well-being (*d*=0.51; *P*<.001), depression (*d*=−0.29; *P*=.008), loneliness (*d*=−0.23; *P*=.04), social support (*d*=0.30; *P*<.001), autonomy (*d*=0.36; *P*=.001), and self-competence (*d*=0.30; *P*<.001; Table 3). Moreover, the proportion of participants with K10 and PHQ-9 scores indicative of severe mental health disorder (K1030) or moderately severe depression (PHQ-915) was significantly lower at follow-up (72% for K10; 53% for PHQ-9) compared with baseline (82% for K10; 58% for PHQ-9; *X*^2^_91_=18.8; *P*<.001 for K10; *X*^2^_91_=19.7; *P*<.001 for PHQ-9).

For those with full access, a secondary analysis revealed that there were significant improvements in psychological distress (*d*=−0.38; *P*<.001), perceived stress (*d*=−0.37; *P*=.01), psychological well-being (*d*=0.38; *P*<.001), loneliness (*d*=−0.33; *P*=.02), social support (*d*=0.25; *P*=.05), and autonomy (*d*=0.50; *P*<.001). Similarly, for those with partial access to MOST+, there were significant improvements in psychological distress (*d*=−0.40; *P*=.03), perceived stress (*d*=−0.55; *P*<.001), psychological well-being (*d*=0.72; *P*<.001), depression (*d*=−0.40; *P*=.03), social support (*d*=0.39; *P*=.03), and self-competence (*d*=0.42; *P*<.001).

### Exploratory Correlations Between System Use, Acceptability Ratings, and Outcome Variables

Given the significantly lower system usage and overall retention rate in participants with partial access compared with those with full access, we reported exploratory correlations between system usage, acceptability ratings, and secondary outcome variables for participants who had full access to MOST+. In terms of system usage and acceptability ratings, there were significant correlations between (1) participants reporting that MOST+ helped them feel better and the number of web-based messages between clinicians and young people (Spearman rho, r_s_=0.53; *P*<.01) and the number of weeks logging in (r_s_=0.42; *P*<.01) and (2) participants reporting feeling more socially connected and the number of comments posted on the newsfeed (r_s_=0.42; *P*<.01) as well as the number of contributions to *Talk it Out* (r_s_=0.32; *P*=.01). With respect to system usage and secondary outcome variables, there were statistically significant correlations between increased relatedness and the number of log-ins (r_s_=0.28; *P*=.03), number of steps completed (r_s_=0.37*; P*<.01), as well as the combined number of *do its* and steps completed (r_s_=0.40; *P*<.01). Increased social support also correlated positively with the number of completed *steps* (r_s_=0.30*; P*=.02) and combined number of *do its* and steps. Moreover, increased strengths usage correlated positively with the number of completed *do its* (r_s_=0.27; *P*=.03). Finally, there was a nonsignificant correlation in the expected direction between lower loneliness and the number of log-ins (r_s_=−0.25; *P*=.06).

## Discussion

To the best of our knowledge, this is the first study to develop and test a multimodal nationwide web-based mental health service for young people experiencing mental ill-health. As such, MOST+ was designed to be an all-in-one digital mental health app merging engaging, evidence-based therapy modules with expert clinician guidance, peer support, social networking, and real-time clinical support. Baseline clinical measures indicated that the majority of participants using MOST+ had moderate (52/157, 33%) to severe mental health conditions (96/157, 61%) and moderate to severe depressive symptoms (130/157, 83%). The results of this study showed that MOST+ was feasible, acceptable, and safe, with all acceptability and safety indicators exceeding the a priori established criteria. The high level of overall satisfaction and perceived helpfulness provided strong support for the relevance of the intervention content and features for help-seeking young people experiencing significant mental ill-health.

Secondary outcome variables showed significant improvements, with small to medium effect sizes, in 8 of the 11 outcomes assessed. These included psychological distress, perceived stress, psychological well-being, depression, loneliness, social support, autonomy, and self-competence. Similarly, the proportions of participants with severe mental health disorders and moderate to severe depression (as indicated by the K-10 and PHQ-9) were significantly lower at follow-up. Although the uncontrolled design of this study did not allow any causal inferences, it was worth noting that there were a number of significant correlations in the expected direction between several indicators of system usage (ie, number of log-ins, number of steps completed, number of *do its* completed, and number of posts made on the social network) and both perceived helpfulness (ie, participants reporting that MOST+ made them feel better and more socially connected) as well as secondary outcome variables (ie, relatedness and loneliness, strengths usage, and social support). These initial findings provide *proof of concept* for MOST+ and lend preliminary support to the potential therapeutic effects of the intervention.

MOST+ was designed as a scalable and efficient online youth mental health service integrating multiple modes of digital therapy, available 24/7, thus catering to individual needs and preferences of young people. The combination of treatment modalities integrated by MOST+ was reported in previous research [38,47,48,89-91]. Specifically, recent studies have found that web e chat can be as effective as face-to-face interventions and even out-perform face-to-face treatment, possibly via increased focus on essential treatment goals [47,48]. Writing in web chats can also have significant advantages, such as enabling users to re-read and reflect on the therapist’s responses and their own emotions and promote a sense of control of the pace, content, and depth of disclosure [48]. Moreover, online chat integrated with a web-based intervention was shown to increase clinical effects compared with web-based support alone [89]. Finally, online peer support showed promise in improving mental health outcomes [38,90], potentially improving intervention adherence and satisfaction [45,92], although some studies found no additional therapeutic effect of peer support compared with traditional online interventions [91]. The patterns of use of MOST+, with different young people interacting with different features (ie, steps, web chat with clinicians, and web-based social network) over different timeframes (ie, one-time usage vs multiple extensions) coupled with the finding that 88% (81/93) of the participants reported that the intervention was relevant to their needs, provide support to this approach. Furthermore, the fact that 41% (64/157) of young people did not request a web chat while using MOST+ indicated that this model could increase the efficiency and scalability of traditional web chat services by reducing their reliance on real-time clinician-delivered web chat. Although promising, the cost-effectiveness of MOST+ against traditional web chat services needs to be established via controlled studies.

With the purpose of ensuring the safety of a (potentially) population-level social network, participants could be granted either full or partial access (excluded access to web-based social networking) to MOST+. This provided an opportunity to examine the differences in satisfaction levels, perceived safety, usage, and secondary outcomes in relation to the level of access to the system. Interestingly, participants with access to the social network reported significantly higher levels of overall satisfaction, showed higher levels of usage of MOST+, and were more likely to be interviewed at follow-up (60/73, 78%) compared with their counterparts in the partial access group (36/84, 43%). Moreover, although loneliness and autonomy improved significantly in the group with full access, these domains remained unchanged in the partial access group. Taken together, these findings suggested that limiting access to the social network may have thwarted the sense of autonomy and the motivation to use the system and remained in the study in those with partial access. Conversely, having full access to the system and social network may lead to increased autonomy and reduced loneliness, irrespective of whether young people posted or not. It must be noted that there were no significant differences in any baseline variables (including clinical severity and basic psychological needs) between those with partial access and those with full access. These findings were in keeping with the self-determination theory, which posited that environments that addressed the basic psychological needs of autonomy (ie, sense that one’s own behavior is freely chosen and of one’s own volition), relatedness (ie, feelings of safety, belonging, and connectedness in their social interactions), and self-competence enhanced intrinsic motivation [93,94] as well as engagement in web-based interventions [95]. Alternatively, it could have been that participants with partial access were more difficult to contact because they were less motivated to use the system and participate in the study in the first place. Taken as a whole, these findings highlighted that, when designing web-based social media–based interventions, safety considerations should be weighed against potential negative effects on engagement or lack of positive effects on social outcomes. For example, future iterations of MOST+ could include an opt-in feature whereby young people decide whether they want to participate in the social network, thus preserving their sense of autonomy. In addition, less burdensome procedures could be introduced to verify age, which accounted for 57% (48/84) of all participants with partial access (eg, uploading an ID card picture via mobile phone as part of the onboarding process or linking their MOST+ account to the existing national web health records to automatically verify age). For those at increased clinical risk, additional safety measures (eg, enhanced automatic and manual monitoring of posts; personalized, detailed information on the purpose and terms of use of the social network) could be implemented to ensure clinical safety while including them in the social network. We successfully implemented this approach in a recent study of a social media–based intervention for young people with elevated suicidal ideation [96].

Web-based social media interventions provide a unique opportunity to address the pervasive rates of social isolation and lack of social support among young people with mental ill-health. For example, young people with psychosis report an average of three lonely days per week [97]. Come adulthood, 75%-94% will experience significant loneliness [98,99]. Unfortunately, recent studies have shown that frequent use of social media among young people is linked to lower self-esteem and increased anxiety, depression, and psychological distress [100,101], with young people with lower well-being being more vulnerable to experience adverse effects when using social media [102]. Against this backdrop, it is essential to develop evidence-based social media–enabled interventions that promote social support while ensuring safety and diminishing harmful consequences. A number of studies from our research lab [42-44,55] and others [39] have shown that carefully designed moderated web-based social media interventions can be safely implemented and are not associated with harmful effects. This study adds to this growing body of evidence by demonstrating that web-based social media interventions can be safely deployed to help-seeking young people via a national web counseling service. That said, the optimal size, functioning, and operations of social media–based interventions remain uncertain. For example, what level of engagement or participation is needed for participants to benefit from these interventions? What is the optimal balance between messages of distress and requests for help (which may lead to contagion [103]) versus messages of hope and positivity (which may alienate some participants [104])? Can web-based social networks provide a safe and transitional *training environment* that leads to real-world improvements? These and other questions will need to be examined in future research using mixed methods, including qualitative studies as well as novel methodologies and analytic techniques such as machine learning and natural language processing.

This study has several limitations. First, the uncontrolled design precluded any causal inferences about the efficacy of MOST+. Second, given that the study was implemented nationally and all assessments were conducted remotely, there was a 40% attrition rate at follow-up, which may have positively biased the results (ie, young people who felt more positively about the intervention may be more likely to be assessed at follow-up). That being said, the reported attrition rate is among the lowest reported by studies evaluating web-based interventions of the equivalent duration via remote assessments (35%-74%) [105-107], and there were no differences in any baseline demographic or clinical variables between those who completed the follow-up assessment and those who did not. Moreover, it is worth noting that MOST+ was not designed to promote sustained engagement, and it did not implement any strategies to foster engagement over time. On the basis of the purpose and function of the eheadspace web counseling service, MOST+ was intended to provide immediate short-term support to help-seeking young people and, where appropriate, redirect young people with long-term needs to youth mental health services. Third, multiple correlations were estimated, which is likely to increase the number of type I errors. Given the exploratory nature of these analyses, we did not adjust for multiple comparisons. Thus, these findings should be considered to be exploratory and interpreted with caution. Fourth, the short-term duration of the study precluded the examination of long-term outcomes. However, this design was considered appropriate, given the purpose of the intervention (ie, addressing immediate psychological distress) and the typically short-term engagement of young people with eheadspace. Finally, the multimodal nature of MOST+ precludes the examination of the effectiveness of the specific elements of MOST+, leading to improved outcomes. Although these research questions were outside the scope of this study, future research adopting novel approaches (eg, rapid A/B testing) will need to determine the optimal combination of features, therapeutic content, and level of usage to improve technological markers (eg, penetration rate, satisfaction, and perceived helpfulness) as well as clinical (eg, symptoms) and social (eg, loneliness, social support) outcomes. Moreover, further research should determine whether MOST+ is effective in fostering help-seeking in, and addressing the needs of, young people with lower socio-economic status or young people at the risk of social exclusion.

### Conclusions and Future Research

The results of this pilot investigation demonstrated that MOST+ is a highly promising and relevant web-based clinical service for young people with clinically significant mental ill-health as it yielded high satisfaction, safety, and perceived helpfulness as well as encouraging improvements in a wide range of clinical and social outcomes. These initial findings provide *proof of concept* for MOST+ and lend support to the multimodal, integrated approach of the intervention.

The effectiveness and cost-effectiveness of MOST+ will need to be established via controlled evaluations addressing the limitations of this study. For example, MOST+ could be implemented as a national service and evaluated through hybrid trial designs that blend components of clinical effectiveness and implementation research [108]. Alternatively, the national deployment of MOST+ would enable fast, well-powered, efficient randomized controlled trials evaluating the effectiveness of the different components of the intervention (eg, dismantling trials) as well as successive iterations of the service. The results and innovations of these trials could be rapidly assimilated into the mainstream service, thereby breaking the current divide and long-term delays between research and clinical implementation [109].

The results from this study indicate that MOST+ is a scalable web-based mental health service that enhances the capacity of traditional web counseling services. Future iterations of MOST+ will incorporate artificial intelligence (AI) and machine learning technologies to further enhance the efficiency of the service (eg, via triaging human support as required) as well as the personalization of the intervention [110]. This could also include chatbots harnessing natural language processing and AI to support participants in finding relevant content within the system, delivering basic therapeutic counseling, and initiating human involvement as needed. Finally, optional built-in video conferencing capabilities may enable faster therapeutic sessions while being able to assess nonverbal cues when necessary.

Finally, in addition to providing mental health support to young people who are not able to access face-to-face care, MOST+ could be integrated with the growing international network of youth mental health services to address wait-list issues, provide continuity of care in between therapy sessions, and offer relapse prevention support after initial treatment response. Meanwhile, MOST+, in its current form, stands to deliver an accessible and scalable web-based mental health service, providing multiple and integrated modalities of web-based support, to cater to the needs of an increasingly growing number of young people with mental ill-health.

## Appendix

Multimedia Appendix 1 Study procedure. MOST+: Enhanced Moderated Online Social Therapy.

Multimedia Appendix 2 Example participant timelines through the Enhanced Moderated Online Social Therapy (MOST+) intervention.

Multimedia Appendix 3 Enhanced Moderated Online Social Therapy (MOST+) intervention safety algorithm.

## Abbreviations

AI: artificial intelligence
CBT: cognitive behavioral therapy
K10: Kessler 10
MOST: Moderated Online Social Therapy
MOST+: Enhanced Moderated Online Social Therapy
NHMRC: National Health and Medical Research Council
PHQ-9: Patient Health Questionnaire-9
SNS: social networking sites
