# Supplementary figures and images for: A Novel Multimodal Digital Service (Moderated Online Social Therapy+) for Help-Seeking Young People Experiencing Mental Ill-Health: Pilot Evaluation Within a National Youth E-Mental Health Service
Source: J Med Internet Res. 2020 Aug 13;22(8):e17155. doi: 10.2196/17155 (PMC7453330; doi:10.2196/17155)

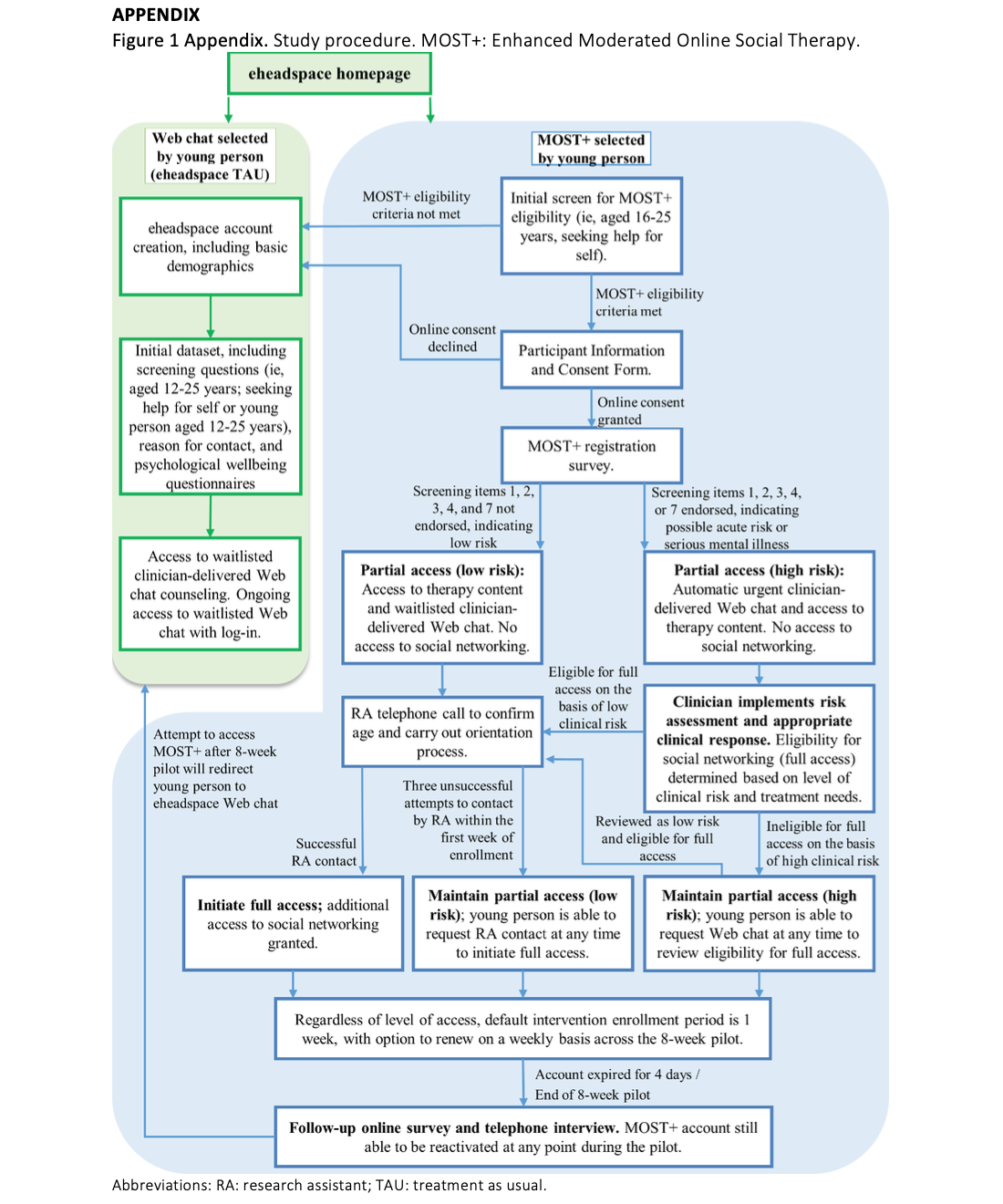

Supplement: Multimedia Appendix 1 [file jmir_v22i8e17155_app1.png]

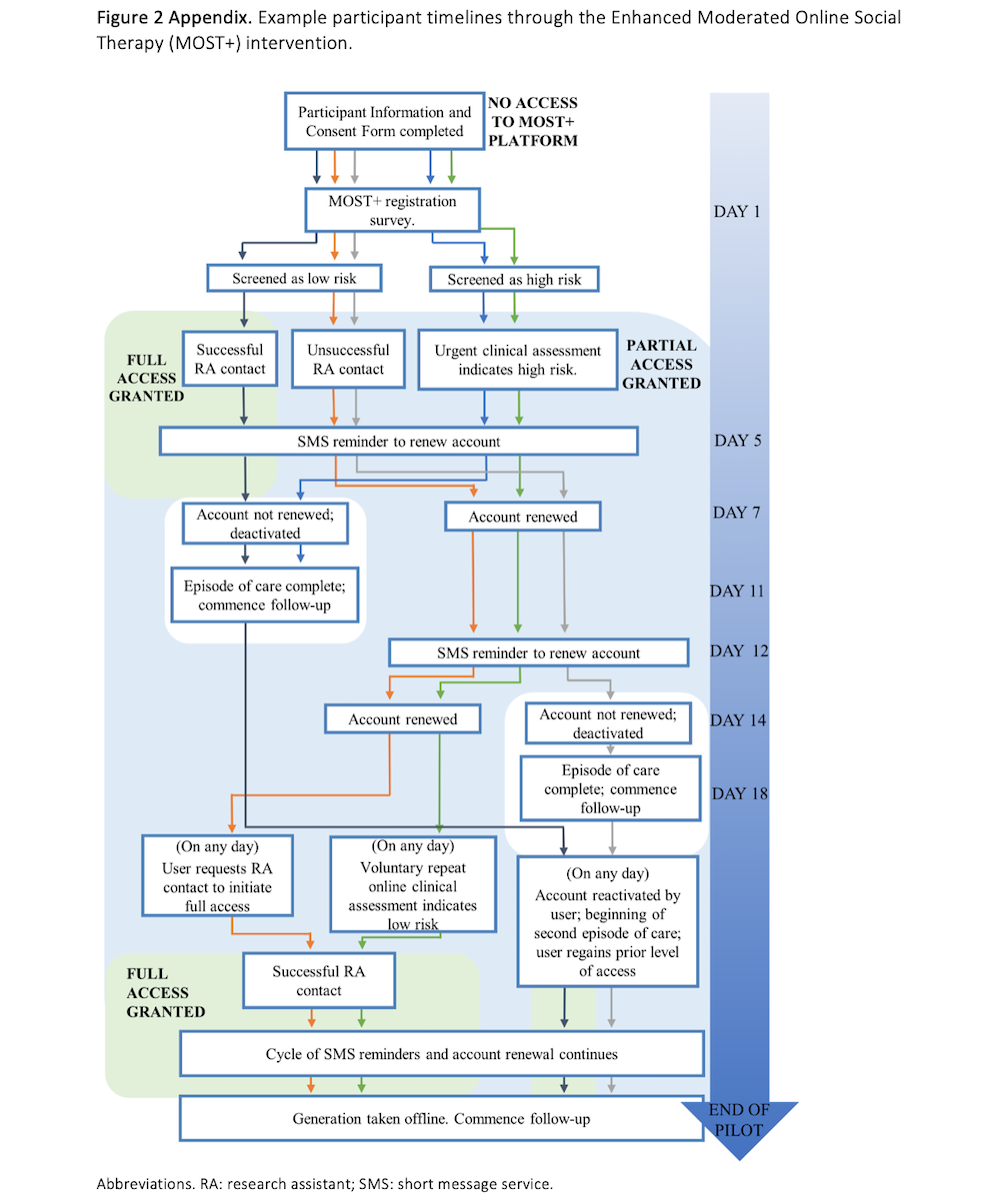

Supplement: Multimedia Appendix 2 [file jmir_v22i8e17155_app2.png]

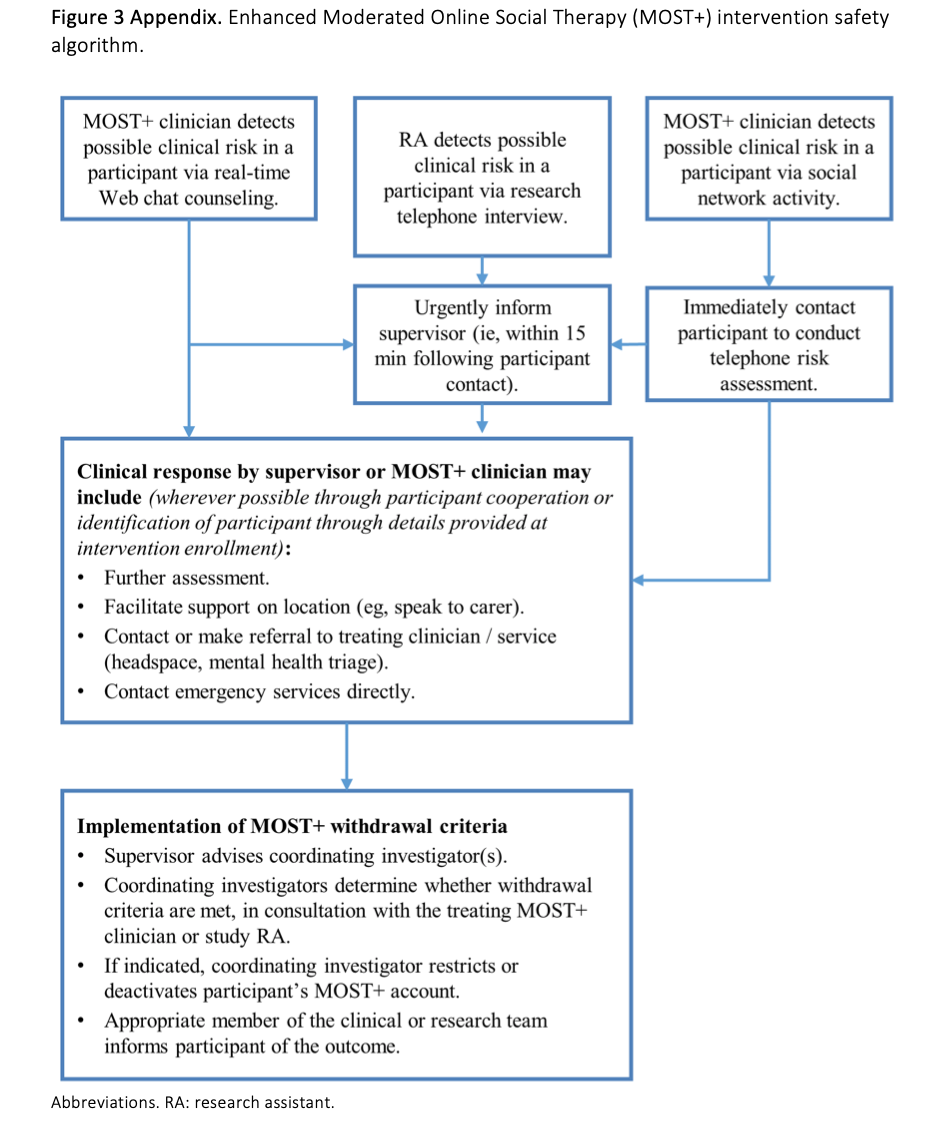

Supplement: Multimedia Appendix 3 [file jmir_v22i8e17155_app3.png]
